# Subclavian Artery Calcification: A Narrative Review

**DOI:** 10.7759/cureus.23312

**Published:** 2022-03-19

**Authors:** Mohamed A Ahmed, Divya Parwani, Anmol Mahawar, Vasavi Rakesh Gorantla

**Affiliations:** 1 Anatomical Sciences, St. George's University School of Medicine, St. George's, GRD

**Keywords:** diagnostic methods, vascular pathology, subclavian steal syndrome, subclavian artery stenosis, subclavian calcification

## Abstract

Subclavian artery calcification (SAC) affects 2% of the population and presents a serious risk of developing into subclavian steal syndrome (SSS). Risk factors for plaque formation of the subclavian artery include diabetes, hypertension, and smoking. While SAC generally presents as asymptomatic, symptoms in severe cases may include numbness, pain at rest, and ischemic gangrene. Patients with severe SSS are at high risk of developing neurological symptoms as a result of vertebrobasilar insufficiency affecting posterior cerebral perfusion. On physical examination, SSS is preliminarily diagnosed from bilateral inter-arm systolic blood pressure discrepancy (>10 mmHg), which can be further confirmed with vascular imaging. Duplex ultrasound (DUS) is a cost-effective and non-invasive baseline technique for visualizing luminal stenosis and quantifying peak systolic velocity (PSV). Computed tomography angiography (CTA) provides high-quality, fast, three-dimensional (3D) imaging at the cost of introducing nephrotoxic contrast agents. Magnetic resonance angiography (MRA) is the safest 3D imaging modality, without the use of X-rays and contrast agents, that is useful in assessing plaque characteristics and degree of stenosis. DUS-assisted digital subtraction angiography (DSA) remains the gold standard for grading the degree of stenosis in the subclavian artery and determining the distance between the puncture site and lesion, which can be carried out in a combined procedure with endovascular management strategies. The fundamental treatment options are surgical and endovascular intervention. Endovascular treatment options include percutaneous transluminal angiography (PTA) for recanalization of the stenosed vessel and permanent balloon stenting to prevent collapse after PTA. Overall, the benefits of endovascular management encompass faster recovery, lower stenosis recurrence rate, and lower incidence of complications, making it the treatment of choice in low-risk patients. Surgical interventions, although more complex, are considered gold-standard treatment options.

## Introduction and background

Subclavian artery calcification (SAC) is the calcium deposition within the arterial walls that leads to stiffened subclavian arteries. Arterial calcification could result from atherosclerotic rupture and plaques calcifications. Calcification could also occur from isolated causes such as smooth muscle calcification in the media layer of medium and large arteries [1]. According to Guzman, 33% of the American population aged above 45 years is diagnosed with arterial calcification when examined by CT [1]. Common risk factors include but are not exclusive to atherosclerosis occlusive disease risks factors such as hypertension, high BMI, and low levels of high-density lipoproteins (HDL) [2]. Coronary artery calcification (CAC) has also been linked to asymptomatic volunteers aged over 50 years. In Arad et al.’s study, a high positive correlation between calcium scores and insulin resistance, high blood triglycerides, low levels of HDL cholesterol, and low-density lipoprotein (LDL) was seen in 1160 asymptomatic volunteers [3]. In their study to evaluate the ethnic influence on CAC levels, Jain et al. examined the prevalence of CAC+ in black and white men and women. However, they found a significant association between cardiovascular risk factors, i.e., “age, smoking, hypertension, and diabetes,” and CAC; however, ethnicity did not show a statistically significant association. They also concluded that ethnic groups with higher cardiovascular risk factors prevalence neither have higher prevalence nor higher odds ratio for CAC positive score [4].

Pathogenesis and pathophysiology

Vascular calcification is the process of abnormal deposition of calcium and phosphates within the blood vessels. It has been linked to atherosclerosis, diabetes, and chronic kidney disease. In addition to valvular calcification, intimal and medial calcifications are the primary forms. While some forms are rare, such as idiopathic infantile arterial calcification, vascular calcification is a common complication in hemodialysis patients [5].

In addition to passive calcium deposition in degenerative tissue, active ossification is seen in vascular calcification in the form of endochondral and intramembranous (non-endochondral) ossifications [6]. Calcific uremic arteriolopathy is another form of calcification seen in hemodialysis [7]. It is hypothesized that vascular smooth muscle responds to the increase of serum phosphate by mineralizing it with calcium in the extracellular matrix. It was also observed that treating isolated vascular smooth muscle with 2 mmol/l of Pi (inorganic phosphate) [8] increased osteocalcin, the calcium-binding protein, and Cbfa-1 mRNAs, an osteoblast transcription factor. Cbfa-1 has also shown the potential to regulate bone matrix deposition rate [9]. The discovery of active vascular calcification strongly suggests the influence of osteotropic hormones in the calcification process. Vascular calcification in uremia could lead to vascular thrombosis or ischemic necrosis [7].

Calcification Location

**Figure 1:**
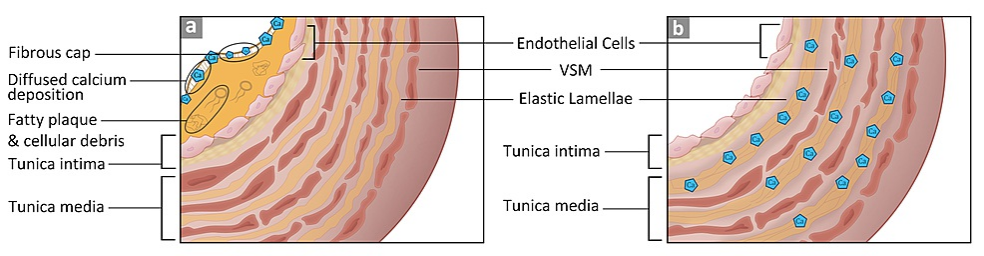
Intimal and medial calcification of blood vessels. Image (A) depicts the intimal calcification with the fibrous cap covering lipid plaque and debris. Image (B) depicts the calcium deposition along the elastin fibers in the tunica media [1,5,6,10]. VSM: vascular smooth muscle.

Vascular walls are made of three layers, tunica intima (innermost), media, and adventitia (outermost). Vascular calcification could occur in either the intimal or the medial layers. Differences in the histological composition between the two layers demarcate the mode of deposition. The tunica intima consists of squamous epithelial cells, basal lamina, and loose connective tissue in the subendothelial layer [5,10]. The tunica media layer has vascular smooth muscle that runs from the internal to the external elastic membranes. These differences dictate the pattern of calcium deposition. If the deposition occurs in the intima layer, it is diffused; if it happens in the media layer, calcium is deposited along the elastin fibers [5]. These two types are depicted in Figure 1.

Clinical Manifestations

Arterial calcification narrows the blood vessels and leads to arterial stenosis. When the subclavian artery is stenosed, there is an increased potential risk of transient ischemic episodes that affect the upper extremities [11]. Other symptoms include intermittent arm claudication, cold upper extremity, and differences in the blood pressure measurements between the two arms. Upon physical examination, a weak unilateral pulse is usually observed with a 10-mmHg difference between the left and the right sides. The affected limb might be cold, and ischemic injury might ensue [11].

Histology of Calcification

Arterial calcification is categorized into four main forms according to their histo-anatomical features. The first class is atherosclerotic or fibrotic calcification. This type is characterized by a fibrous cap covering the fatty plaque [12], which causes the accumulation of cellular debris and the recruitment of macrophages and T lymphocytes. The calcium deposition in this type is associated with lipoproteins. The second type is particular to cardiac valve leaflets. Increased stress on the leaflets triggers an inflammatory state that is characterized by inducing intramembranous-like calcium deposition. The valvular fibrosa becomes susceptible to fatty expansion. The third type is the medial artery calcification, where the tunica media layer undergoes ossifications. In this type, calcification happens at the matrix fibrils. Lastly, vascular calciphylaxis describes general soft tissue calcification due to an overwhelming amount of calcium phosphates. When the serum's calcium pyrophosphate exceeds 60 mg2/dl2, precipitation occurs in the soft tissue. This type is mostly seen in end-stage renal disease and tumor lysis due to increased serum calcium phosphates [6].

Symptomatic vs. Asymptomatic

In most cases, subclavian artery stenosis (SAS) is diagnosed accidentally when the blood pressure difference between the right and left upper extremities is noted or through an imaging study of the carotid or coronary artery. Some SAS cases are discovered during coronary artery bypass graft (CABG) since the plaque hinders the catheter from traversing the subclavian artery [13]. The most common complication of subclavian stenosis is subclavian steal syndrome (SSS), where the ipsilateral vertebral artery supplies the subclavian artery. This reversal flow of blood bypasses an occlusion or severe stenosis in the region proximal to the subclavian and shunts the blood from the contralateral vertebral artery [14]. In their study, Labropoulos et al. examined 7881 patients for SSS and 4.2% were found to have a blood pressure difference >20-30 mmHg between the right and the left upper extremities [15]. Out of the 4.2%, only 77% had SSS. Most of the cases (82.3%) had SSS on the left side. The retrograde flow “was complete in 61%, partial in 23%, and absent in 16%.”

Diagnostic Methods

SAS affects 2% of the population and presents as asymptomatic in most cases [16]. Cervical discopathy is a common misdiagnosis of SAS symptoms [16]. Therefore, physical examination and diagnostic imaging are relevant tools in the reliable and cost-effective detection of cardiovascular diseases. According to Hong, left SAS presents with coolness, numbness, pain at rest, and gangrenous discoloration of the left upper extremity [17]. Left SAS has a higher incidence of stenosis compared to the right subclavian artery. Bilateral inter-arm systolic blood pressure measurement between both upper extremities is the next step; a discrepancy of >10 mmHg is an excellent indicator of cardiovascular mortality. Atherosclerotic plaques are found in the proximal segment of the subclavian artery, causing compensatory retrograde flow in the vertebral artery resulting in vertebrobasilar insufficiency (VBI). Moreover, reduced perfusion of downstream brachial artery presents as upper arm claudication. Devices like pacemakers and defibrillators make visualization of the subclavian artery a challenge, which brings the need for angulation in imaging modalities [18]. The North American Symptomatic Carotid Endarterectomy Trial (NASCET) parameters are used to quantify the degree of stenosis with the formula (1-A/B)*100, where A represents the narrowest stenotic lumen and B represents the largest diameter upstream to the stenosed section of the lumen [16].

Duplex Ultrasound

Duplex ultrasound (DUS) is the readily available baseline test for diagnosing SAS, as it provides low-cost visualization of the anatomy and blood flow in the vessel [19]. According to Kablak-Ziembicka, convex probe DUS (3-5 MHz) was the only method of ultrasound useful for viewing subclavian artery obstruction in 64.4% of 118 patients [20]. DUS has a sensitivity of 94 for 70% stenosis and 73 for 50% stenosis; additionally, the specificity was 92 for 70% stenosis and 91 for 50% stenosis [16]. Furthermore, DUS provides additional information about the obstruction, such as flow direction, turbulence, spectral changes, and peak systolic velocity (PSV) (in cases of high-grade stenosis, PSV: 4.4 ± 1.2 m/sec; range: 2.2-6.5 m/sec) [20]. The application of DUS falls short in analyzing proximal and deeper areas of the subclavian artery, as well as the accurate degree assessment of the stenosis [21].

Computed Tomography Angiography and Magnetic Resonance Angiography

Computed tomography angiography (CTA) provides better visualization for evaluating the degree and type of stenosis in subclavian arteries. The recent advancements in fine focal spot size (FFSS) technique in CTA reduces calcification artifacts (U: 2040.50, p < 0.001, r: 0.564) and increases spatial resolution (U: 48238.50, p < 0.001, r: 0.556) standard focal spot size (SFSS), which provides greater efficacy in analyzing the degree of stenosis [22]. The limitation of CTA is that it exposes the patient to radiation and high proton intensity leading to heat generation, which makes the technique unsuitable for use in patients with a history of renal diseases [20]. FFSS (0.5 x 1 mm) utilizes a 16.2% lower radiation dose than SFSS (1 x 1 mm), making it a safer and faster choice in emergent situations [19]. Magnetic resonance angiography (MRA) provides a better contrast to visualize the flow of blood as compared to surrounding tissues and arterial lumen. Earlier studies concluded that MRA falls short in being able to differentiate between complete occlusion and severe stenosis, which is no longer the case with advancements in technology [21]. MRA now not only provides non-invasive imaging of the degree of stenosis but is also helpful to analyze plaque characteristics [23]. The application of MRA must be closely monitored in patients; they must have a compatible pacemaker or cardioverter-defibrillator device. Both CTA and MRA provide an anatomical cross-sectional visualization useful in determining the location, length, and plaque morphology [19].

Digital Subtraction Angiography

Digital subtraction angiography (DSA) is accurately able to quantitatively evaluate the degree of stenosis as a function of pressure gradient [18]. A systolic pressure gradient of 15-20 mmHg indicates severe stenosis and CTA provides real-time fluid flow characteristics useful for measuring the systolic pressure drop across the subclavian artery [18,21]. This technique is considered overall safe but has a risk of complications such as upper extremity ischemia and embolism in 0-1.7% of patients [18].

Current management strategies

Medical Management

The ultimate objective of medical care in patients with SAC is to reduce progression to atherosclerotic diseases and cardiovascular complications. Usai et al. observed that the group of patients who underwent surgery had a reduced number of interventions post-surgery as opposed to the group of patients who sought endovascular treatment. Although there was no significant difference observed in the survival rates among the two groups, it was observed that after 98 months, the re-intervention-free survival (RFS) rate was 95% after surgical treatment compared to 54% after endovascular treatment (P = 0.0002). It was concluded that despite the low morbidity and mortality rates associated with endovascular treatment, surgical intervention remains the benchmark for subclavian artery arteriosclerotic disease [24-26].

Modifiable risk factors such as hypertension, smoking, and increased glycemic levels are associated with an increased risk of developing calcifications in the subclavian, carotid as well as aortic arteries [26]. Studies conducted by Prasad et al. found that SAC was also linked to decreased diastolic blood pressure (DBP) and a widened pulse pressure (PP) in addition to the aforementioned risk factors [26].

Atherosclerosis being a leading risk factor in developing SAC can be managed by administering statins to reduce cholesterol levels as well as angiotensin-converting enzyme (ACE) inhibitors in lowering blood pressure. Antiplatelet medications such as aspirin can also be used to reduce blood clotting in these individuals [27].

Endovascular interventions are first-line treatment options in asymptomatic patients with SAC. Additionally, patients who have unsuitable anatomy of major arteries may opt for endovascular procedures to prevent damage to their vessels, which may be exacerbated by surgical interventions [24,28]. The procedure can be conducted either by percutaneous angioplasty (PTA) or balloon stenting. Percutaneous balloon stenting is a preferred mode of managing patients with SSS as the combined treatment provides long-term patency rates as well as reduces the chances of restenosis occurrence [29,30]. According to the guidelines issued by the European Society of Cardiology in 2018 as well as studies conducted by Chatterjee et al., percutaneous balloon stenting is a favorable option for managing symptomatic as well as asymptomatic patients with SSS [30,31]. Symptoms of SSS include intermittent pain in the upper limbs, PP discrepancies in both arms as well as neurological manifestations such as lightheadedness and syncope [11,32]. The procedure involves dilating the blood vessel using balloon angioplasty followed by catheter insertion to place a stent in the vessel to prevent it from collapsing [33,34].

**Figure 2:**
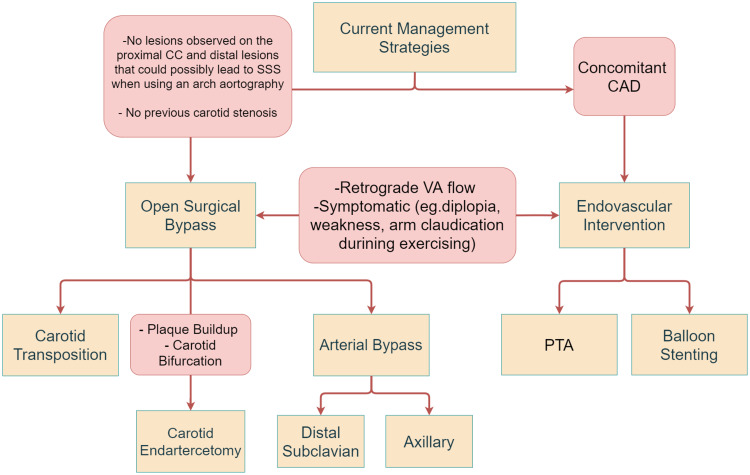
Current management strategies based on the stenosis severity and patients history. CC: common carotid; SSS: subclavian steal syndrome; VA: vertebral artery; CAD: coronary artery disease; PTA: percutaneous transluminal angioplasty [40].

Open surgical bypass is recommended for patients with no comorbidities [35]. While there are minimally invasive techniques available that provide long-term patency rates and decrease the likelihood of blockage, surgical interventions remain the gold standard treatment for managing patients [24,25]. Surgical procedures can be carried out either by subclavian carotid transposition (SCT) or CABG. Patients undergoing SCT commonly present with symptoms of VBI, including numbness and diplopia [36]. SCT is a favorable method in patients who have a lesion in the proximal portion of the subclavian artery [37]. Lastly, carotid endarterectomy (CEA) is a suitable treatment option for patients with plaque deposition in their blood vessels as well as patients with carotid bifurcation [38]. The procedure can be performed under general or local anesthesia after which an incision is made in the neck to access the vessel with plaque buildup. The artery is opened up, after which a shunt may be used to allow continuous blood supply to the brain [39]. Management options are discussed in Figure 2.

## Review

Discussion

With medical advancements, methods such as endovascular intervention are less invasive and deemed suitable for patients with concomitant coronary artery disease; however, surgical interventions remain the gold standard for treatment of subclavian artery atherosclerotic disease (SAAD) [24]. Open surgical bypass remains a suitable treatment option for patients who do not have a history of carotid stenosis and do not have any apparent lesions on the carotid vessels, which could lead to SSS when using an arch aortography [28]. Open surgical bypass can be performed via SCT or CEA, or CABG. SCT is used as a suitable treatment in patients with symptoms such as upper limb ischemia, digital embolization, intermittent claudication as well as neurological manifestations [37]. Studies conducted by Duran et al. and Salman et al. suggest that SCT can be performed when the lesion is proximal to the subclavian artery and the carotid artery is free from any infections [36,37]. An incision is made above the clavicle after which a surgical dissection is made between the sternocleidomastoid (SCM) as opposed to the dissection made while inserting a bypass graft, which is done laterally to the SCM [41]. Additionally, care must be taken while making sub-wide platysma flaps to prevent injury to the external jugular vein. The subclavian artery is transected distal to the origin of the vertebral artery to preserve cerebral blood flow, and in the meantime, clamps can be placed on the subclavian, internal mammary, and thyrocervical trunks to prevent blood loss. The subclavian artery is transected proximal to the vertebral artery and an arteriotomy is made in the carotid artery to anastomose with the subclavian artery [37,41]. Due to the absence of insertion of any foreign material during surgery, SCT is proven to be advantageous as it reduces the risk of infections and emboli formation [37]. However, due to the invasiveness of this procedure, care must be taken to avoid injury to the pleural space [41]. This can be ensured by making the opening incision very close to the wall of the artery [41].

CEA is the preferred surgical option in patients with plaque buildup in their arteries as well as carotid bifurcation [28]. Carotid stenosis can exacerbate symptoms such as dizziness, intermittent claudication, and other neurological symptoms if left untreated. To prevent the consequences of subclavian and carotid stenosis, CEA is recommended [42]. Studies conducted by Risty et al. concluded that concomitant use of CEA with SCT or carotid-subclavian bypass (CSB) increases perioperative risk by 4.73%, whereas the use of SCT or CSB alone has a perioperative risk of 0.32% (P < 0.01) [42]. Most patients improve by CEA alone, which is usually performed under a cervical block. However, if symptoms persist, SCT or CSB may be necessary [42]. The procedure is usually begun by general or local anesthesia, after which an incision is made in the neck in the area of the carotid artery. A catheter is placed in the artery to allow blood flow to the brain. After the plaque is removed, the artery is sutured up [43]. Alternatively, a shunt may also be used to redirect the blood to the brain while the plaque is being removed from the artery [40].

CABG is another surgical option that can be used in patients with symptomatic subclavian stenosis. It is a suitable treatment option in patients that have an injury proximal to the subclavian artery with no signs of hemorrhage [30]. CABG lowers the chances of restenosis as opposed to percutaneous interventions, which have an increased likelihood to do so [44]. CABG commonly involves usage of the left internal mammary artery (LIMA) as it reduces the likelihood of long-term occlusions [44]. CSB using polytetrafluoroethylene (PTFE) grafts is a suitable method in good-risk patients [45]. Literature found by Takach et al. suggests that CSB provides long-term patency rates as well as an overall reduction in morbidity and mortality rates during operation [46]. A horizontal incision is made in the neck, followed by an arteriotomy in the common carotid artery to anastomose it with the subclavian artery [38]. While CSB is proven to be durable, it should only be used as a treatment option in younger patients or patients who need revascularization after a myocardial infarction due to its adverse impact in the long term [46].

In addition to surgical intervention, percutaneous transluminal angioplasty (PTA) is one of the best choices for patients due to the simplicity of the procedure. PTA is a less invasive endovascular treatment for recanalizing the subclavian artery and improving symptoms. According to Kablak-Ziembicka, PTA is a straightforward procedure with faster recovery times compared to CABG [20]. A 3.6% combined stroke and death complication rate has been noted for PTA [31]. Balloon stenting alongside PTA has been found to decrease stenosis recurrence (P = 0.004, 95% CI) [38]. PTA involves a catheter-guided balloon insertion inflated at the site of maximum stenosis to increase the luminal diameter. Balloon stent operates on the same principle except the inflated balloons are left inserted permanently to prevent the collapse of stenosed vessels.

One study conducted by Sixt et al. found that “stent-assisted angioplasty” has a higher patency rate than PTA alone (89% and 75%, respectively) [47]. One common complication of endovascular treatment is the formation of emboli in the posterior circulation. To avoid such a complication, Yamazaki et al. devised the “Balloon Switching” technique in which a two balloon system is installed; the first balloon (of guiding catheter) is placed at the junction of subclavian and vertebral arteries, this will occlude both and prevent the formation of emboli when the second (of the expandable stent) balloon is inflated [48]. This is followed by the deflation of the first balloon of the guiding catheter. This step will free some of the atherosclerotic debris, but since the second balloon is occluding the vertebral artery, the debris is manually aspirated through the balloon-guiding catheter [48]. This method has the advantage of preventing embolism formation in the vertebral artery. Another technique that was developed to tackle the same issue is the “balloon-guiding catheter and the pull-through technique.” Yamamoto et al. reported the use of this technique to prevent embolism formation in the vertebral artery [49]. After a hybrid PTA and stenting procedure, the rate of recurrence is 41% higher in continuous as compared to intermittent SSS. Due to 3.4% recurrence of coronary SSS after CABG, it is recommended that diagnostic PTA be performed preceding CABG [50]. Management options of SAC and stenosis are summarized in Tables 1, 2.

**Table 1:** Current interventions in the management of subclavian artery calcification. EEC: electroencephalogram; PTA: percutaneous transluminal angioplasty; SCT: subclavian carotid transposition; VBI: vertebrobasilar insufficiency; SSS: subclavian steal syndrome; SAS: subclavian artery stenosis; CC: common carotid; CABG: coronary artery bypass graft; CEA: carotid endarterectomy.

Intervention	Findings	Description	Conclusion
1. Endovascular intervention	Endovascular treatment is commonly used as first-line management for asymptomatic patients and for patients with suitable anatomical positioning of the adjacent arteries to preserve their intactness [24,28].	It is mainly classified into two types: (i) percutaneous transluminal angioplasty (PTA) and (ii) stenting. The two procedures can be combined and are known as percutaneous balloon stenting.	Endovascular interventions are preferred over open surgery due to the invasiveness of surgical procedures. However, tortuosity of the subclavian artery and the proximity to the vertebral artery may impose a challenge for deploying the balloon stent.
(i) Percutaneous balloon stenting	According to the European Society of Cardiology Guidelines of 2018, percutaneous balloon stenting was the preferred method of management for patients with SSS [31]. Patients with SSS could be symptomatic or asymptomatic. Symptoms are often described as syncope, arm claudication, and dizziness due to a difference of >15 mmHg in systolic blood pressure [31].	This minimally invasive procedure includes using balloon catheters that should be dilated intermittently to decrease the complications [33]. After inserting a catheter in the blood vessel, care must be taken to place the stent carefully in the area of occlusion. Insertion of a stent allows the occluded artery from collapsing [34].	Literature suggests that stenting after (PTA) has shown to have superior results than PTA by itself [29]. Studies conducted by Chatterjee et al. showed that the usage of stents post-PTA had shown to have longer patency rates as well as a minimal likelihood of long-term complications [30].
2. Open surgical bypass	Recommended for patients with few or no comorbidities [35]. It is also recommended for patients with injury to the subclavian artery due to embolization from distal vessels [24]. Stenosis of greater than 10 cm or occlusions longer than 2 cm would suggest surgical invasion [35].	It is mainly classified into two types: (i) carotid transposition and (ii) arterial bypass	Surgical treatment includes revascularizing the subclavian artery. The most common donor artery is the CC due to its high patency rate. Resolving coexisting carotid stenosis should be prioritized.
(i) Subclavian carotid transposition (SCT)	In a study done by Duran et al., it was found that the majority of patients undergoing SCT presented with symptoms such as upper limb pain, tingling sensations as well as other neurological manifestations such as vertigo and visual disturbances to name a few [36].	The surgical procedure is initiated by regional anesthesia as well as cerebral monitoring using electroencephalogram (EEG). After making a precise incision of 5 cm just 2 cm above the clavicle, the subclavian artery is resected post-stenotically and joined to the ipsilateral carotid artery. To avoid distortion of the vertebral artery, the subclavian artery must be rotated carefully when anastomosed with the common carotid artery [36]. This method is suitable in instances where the occlusion on the subclavian artery is proximal, allowing easy maneuverability of the distal portion of the subclavian artery [37].	There are several advantages of SCT including minimal invasion as opposed to other methods such as a carotid-axillary bypass. Additionally, the anatomical location of the carotid and subclavian arteries make it a suitable site for anastomosis. Due to diminished use of grafts in SCT, there is minimal risk of infections and other graft-related complications [36].
(ii) CABG	This method is used in patients with symptomatic subclavian stenosis. Bypass grafting is indicated in patients with symptoms such as upper limb claudication or vertebrobasilar insufficiency (VBI) characterized by tinnitus, vertigo, diplopia, numbness, etc.	A longitudinal arteriotomy in the common carotid artery can be made to anastomose it with the subclavian artery. To access the subclavian artery, a transverse supraclavicular incision must be made followed by transecting the subclavian artery distal to the occlusion and ultimately ligating it with the common carotid artery [37].	While the main aim of CABG is to supply the cardiac muscles, the CABG blood flow will be reversed in SAS due to reduced pressure. The coronary artery is connected to the distal segment of the subclavian or the axillary artery.
(iii) CEA	This method is used in patients who have occluded arteries due to the buildup of plaque. It is also a preferred treatment in patients with carotid bifurcation [38].	An incision is made in the neck to access the arteries. A shunt is used to deflect the blood to the brain during the procedure, followed by an incision in the artery to remove the plaque [39].	CEA is a treatment of choice in patients who have plaque buildup in their vessels as well as carotid bifurcation [40].

**Table 2:** Pathogenesis and pathophysiology, diagnostic methods, and management of subclavian artery stenosis. CAC: coronary artery calcification; DU: Doppler ultrasound; ER: endovascular repair; PSV: peak systolic velocity; FFSS: fine focal spot size; CTA: computed tomography angiography; OSR: open surgical repair; PTA: percutaneous transluminal angioplasty; RFS: re-intervention-free survival; SAAD: subclavian artery atherosclerotic disease; SAC: subclavian artery calcification; SCT: subclavian carotid transposition; SFSS: standard focal spot size; SSS: subclavian steal syndrome; SAS: subclavian artery stenosis.

Author	Country	Study population	Findings	Conclusion
Jain et al. (2004) [4]	United States	1289	The study was examining the prevalence of CAC scores in African-Americans and Caucasians. CAC scores increased with age in all “ethnicity-gender groups.” The CAC scores were higher in men than women across the two ethnicities. Black women had a CAC+ of 29% as compared to white women (23%), but P = 0.21. The odds ratio of CAC+ among blacks and whites was found to be 1.2 (95% CI: 0.94-1.54).	Even though the prevalence of cardiovascular risk factors (mean blood pressure and mean blood glucose) was significantly higher in the black population, no significant statistical differences were found between black and white men and women for coronary artery calcium (CAC) levels when using weighted data. These findings rule out ethnicity contribution to high CAC. Similarly, there is no difference in the odds ratio of CAC+ between the two ethnicities.
Labropoulos et al. (2010) [15]	United States	7881	Upper extremity blood pressure discrepancy >20-30 mmHg was observed in 4.2%, with a 77% prevalence of subclavian steal syndrome (SSS). When the difference was >30 mmHg.	Most SSS patients are asymptomatic and cases are discovered accidentally while performing duplex scanning. A blood pressure difference of >40-50 is recommended to estimate the severity of the disease.
Hong et al. (2016) [17]	China	20	A treatment of PTA, stenting, and either coronary artery bypass on the same day (hybrid) or four weeks later (staged) was compared between 20 patients. The median hospital stay was nine days for both groups (P = 0.299), mortality was 0 and 3 months, and postoperative angiography showed no notable difference in recurrent left SAS (P = 0.762).	A hybrid approach was concluded to be better for treatment. Further studies need to be carried out to evaluate long-term implications.
Mousa et al. (2017) [16]	United States	117	The values for different parameters evaluated such as PSV, stenosis, area under the curve, selectivity, and sensitivity were found to be 212.6 ± 110.7 cm/s (>240 cm/s for >70% stenosis), 25.8% ± 28.2%, 0.94 (CI: 0.91-0.97), 82.5% (>70), and 90.9% (>70), respectively.	The Youden Index cutoff point was found to be PSV > 240 cm/s, which is indicative of severe SAS. If a positive predicted value was to be used as the control variable, then the cutoff point would be >280 cm/s.
Kablak-Ziembicka et al. (2007) [20]	Poland	118	DU was able to detect 123 lesions on 118 patients. A diagnosis of SAS was made based on pulse-Doppler flow, detected using DU and SSS. The elevated value of the parameter PSV (4.4 ± 1.2 m/sec) was useful in diagnosing the remaining patients. Results of the diagnosis were compared with data from aortic arch angiography and found to have no significant differences.	DU is useful and accurate in determining the presence and severity of subclavian stenosis.
Oh et al. (2019) [22]	United Kingdom	91	Comparative study of 91 patients between SFSS (2.35 sV, 1 x 1 mm) and FFSS (1.97 sV, 0.5 x 1 mm) indicated reduction in calcification artifact (U = 2040.50) and increased vessel wall clarity (U = 48238.50). Aortic pulsation artifact did not significantly differ between the two techniques (U = 958.50).	FFSS in CTA yields higher resolution and low artifact imaging, which may improve the assessment of the degree of stenosis and calcification plaque.
Usai et al. (2020) [25]	Germany	110	Patients who underwent surgical repair had a 95% better RFS compared to 54% seen in the endovascular group (P = 0.0002).	Despite the advancements in the treatment of SAAD, surgical repair remains the optimal method of management.
Prasad et al. (2011) [26]	United States	1387	of the participants, 31.7% had SAC, with a statistically significant older population. Hypertension was not different among SAC+ and SAC- patients, but there was a significant difference in the diastolic blood pressure. There was also an increased arterial calcification, such as carotid = 24.6%, coronary = 60.3%, renal = 18.2%, iliac = 58.9%, and aortic vasculature = 62.5%.	Aging, hypertension, and smoking are strong associates of vascular calcification. The aorta, coronary, and iliac arteries are the top three affected vasculatures.
Chatterjee et al. (2013) [30]	United States	544	The study involved the usage of various databases such as PubMed, CINAHL, Embase, and CENTRAL to identify the efficacy of PTA by itself and stent placement after PTA. The records were screened for duplication and a total of 14 were identified. A total of eight studies were finalized. No significant bias was detected using a funnel plot as well as an Egger test (P = 0.11).	The use of stents after PTA showed significantly better results than PTA by itself. No significant complications were associated with the procedures (P = 0.004; 95% CI: 2.37 (1.32-4.26)).
Benhammamia et al. (2020) [35]	Italy	68	Retrospective data were collected for subjects who underwent OSR and ER between 2001 and 2018. After a 30-day interval, both groups showed equal rates in improvements with neither neurological symptoms nor cardiac events. The long-term patency rate observed after seven years was higher in the ER group (93.4% + 4.5%) compared to the OSR group, which was found to be 76.9% + 11.7% (P = 0.02).	SAAD showed similar outcomes when treated with both OSR as well as ER.
Duran et al. (2015) [36]	Germany and Luxembourg	126	A group of 126 subjects was monitored for SCT between 1995 and 2013. After a period of 30 days, the primary and secondary patency rates were 96% and 100%, respectively. A follow-up after 53.8 months revealed a long-term patency rate of 96.3%.	SCT is a long-lasting and effective treatment for symptomatic subclavian stenosis. The results of the patency rates are superior to those found with endovascular interventions; however, the risk of local complications cannot be overlooked.

## Conclusions

SAC is one of the most common complications of atherosclerosis. Most of the affected patients are asymptomatic, and the treatment of choice depends on the severity of each case. Patients with ischemic symptoms require immediate intervention. DUS is the preliminary testing technique; the diagnosis is confirmed by either CTA or MRA. Antiplatelet therapy should be used in patients regardless of the disease progression to control underlying risk factors. Treatment options are classified as open surgical bypass or endovascular intervention. While surgical interventions are not recommended for asymptomatic patients, mild symptomatic patients are recommended to undergo endovascular treatment to revascularize the lesion. Percutaneous balloon stenting is preferred over percutaneous transluminal angioplasty, as it reduces stenosis recurrence. Patients with severe occlusions are advised to undergo surgical bypass to ensure adequate blood supply through the distal subclavian segment. SCT is preferred in patients with severe vertebrobasilar insufficiency, and CEA is preferred in patients who have plaque accumulation in their arteries. Lastly, surgical bypass is recommended in patients with no comorbidities. While surgical interventions are the gold standard for treating SAC, endovascular interventions are the preferred treatment choice for patients with SAC.
